# *N*-(1,3,4-Oxadiazol-2-yl)Benzamides as Antibacterial Agents against *Neisseria gonorrhoeae*

**DOI:** 10.3390/ijms22052427

**Published:** 2021-02-28

**Authors:** George A. Naclerio, Nader S. Abutaleb, Marwa Alhashimi, Mohamed N. Seleem, Herman O. Sintim

**Affiliations:** 1 Chemistry Department, Institute for Drug Discovery, Purdue University, West Lafayette, IN 47907, USA; gnacleri@purdue.edu; 2 Department of Comparative Pathobiology, Purdue University College of Veterinary Medicine, West Lafayette, IN 47907, USA; nabutale@purdue.edu (N.S.A.); alhashim@purdue.edu (M.A.); 3 Department of Biomedical Sciences and Pathobiology, Virginia-Maryland College of Veterinary Medicine, Virginia Polytechnic Institute and State University, Blacksburg, VA 24060, USA; 4 Purdue Institute of Inflammation, Immunology, and Infectious Disease, West Lafayette, IN 47907, USA

**Keywords:** *Neisseria gonorrhoeae*, 1,3,4-oxadiazole, antibiotic, antimicrobial resistance

## Abstract

The Centers for Disease Control and Prevention (CDC) recognizes *Neisseria gonorrhoeae* as an urgent-threat Gram-negative bacterial pathogen. Additionally, resistance to frontline treatment (dual therapy with azithromycin and ceftriaxone) has led to the emergence of multidrug-resistant *N. gonorrhoeae*, which has caused a global health crisis. The drug pipeline for *N. gonorrhoeae* has been severely lacking as new antibacterial agents have not been approved by the FDA in the last twenty years. Thus, there is a need for new chemical entities active against drug-resistant *N. gonorrhoeae*. Trifluoromethylsulfonyl (SO_2_CF_3_), trifluoromethylthio (SCF_3_), and pentafluorosulfanyl (SF_5_) containing *N*-(1,3,4-oxadiazol-2-yl)benzamides are novel compounds with potent activities against Gram-positive bacterial pathogens. Here, we report the discovery of new *N*-(1,3,4-oxadiazol-2-yl)benzamides (**HSGN-237** and **-238**) with highly potent activity against *N. gonorrhoeae*. Additionally, these new compounds were shown to have activity against clinically important Gram-positive bacteria, such as methicillin-resistant *Staphylococcus aureus* (MRSA), vancomycin-resistant enterococci (VRE), and *Listeria monocytogenes* (minimum inhibitory concentrations (MICs) as low as 0.25 µg/mL). Both compounds were highly tolerable to human cell lines. Moreover, **HSGN-238** showed an outstanding ability to permeate across the gastrointestinal tract, indicating it would have a high systemic absorption if used as an anti-gonococcal therapeutic.

## 1. Introduction

Drug-resistant bacterial infections have become a serious global threat. *Neisseria gonorrhoeae* is a Gram-negative bacterial pathogen which causes gonorrhea, a sexually transmitted infection (STI) [1]. *N. gonorrhoeae* infects a variety of mucosal surfaces (i.e., the urethra, endocervix, pharynx, and rectum) [2] and, if left untreated, can cause drastic complications, such as pelvic inflammatory disease, ectopic pregnancy, and increased susceptibility to HIV infections [3]. The Centers for Disease Control and Prevention (CDC) considers *N. gonorrhoeae* an urgent threat, as it accounts for 550,000 infections per year and $133.4 million dollars in medical costs in the United States alone [4]. Globally, *N. gonorrhoeae* is also devastating. The World Health Organization (WHO) listed *N. gonorrhoeae* as a priority 2 (high) pathogen, as it is has caused 87 million new cases as well as an estimated total treatment cost of $5 billion dollars [5,6,7,8]. 

Efforts to develop novel antibiotics against urgent threat pathogens, especially *N. gonorrhoeae*, have intensified [9]. For instance, *N. gonorrhoeae* is now resistant to former front-line therapies such as penicillin, fluoroquinolones, and cefixime, which are now deemed ineffective as treatment options [10]. This increased resistance rate prompted a global health scare, leading the CDC to recommend treating *N. gonorrhoeae* with dual therapy involving ceftriaxone and azithromycin [1,11]. Yet, resistance to this dual therapy has been reported, leading to the rise of multidrug-resistant *N. gonorrhoeae* (commonly referred to as super gonorrhea) [2]. To make matters worse, no new classes of antibiotics to treat *N. gonorrhoeae* have been FDA-approved over the last two decades, warranting the public health concern that once easily treated gonorrhea infections will soon become deadly [12,13,14]. Therefore, the rise in multidrug-resistant *N. gonorrhoeae* infections necessitates intense research efforts to identify and develop new antibiotics.

Our program focuses on the development of *N*-(1,3,4-oxadiazol-2-yl)benzamides to treat drug-resistant bacterial pathogens [15,16]. We recently reported the discovery of trifluoromethylsulfonyl (SO_2_CF_3_), trifluoromethylthio (SCF_3_), and pentafluorosulfanyl (SF_5_) containing *N*-(1,3,4-oxadiazol-2-yl)benzamides that exhibited potent antibacterial activities against clinically important Gram-positive bacterial pathogens [17]. These agents were found to be active against clinical isolates of drug-resistant Gram-positive bacteria, were non-toxic to mammalian cells, and effectively reduced the burden of intracellular methicillin-resistant *Staphylococcus aureus* (MRSA) [17]. Here, we describe a new generation of *N*-(1,3,4-oxadiazol-2-yl)benzamides with potent activity against *N. gonorrhoeae.* The antibacterial activity against *N. gonorrhoeae*, cytotoxicity against mammalian cells, and bi-directional Caco-2 permeability were investigated.

## 2. Results and Discussion

### 2.1. Synthesis and Antigonococcal Activity of N-(1,3,4-oxadiazol-2-yl)benzamides:

**Figure 1:**
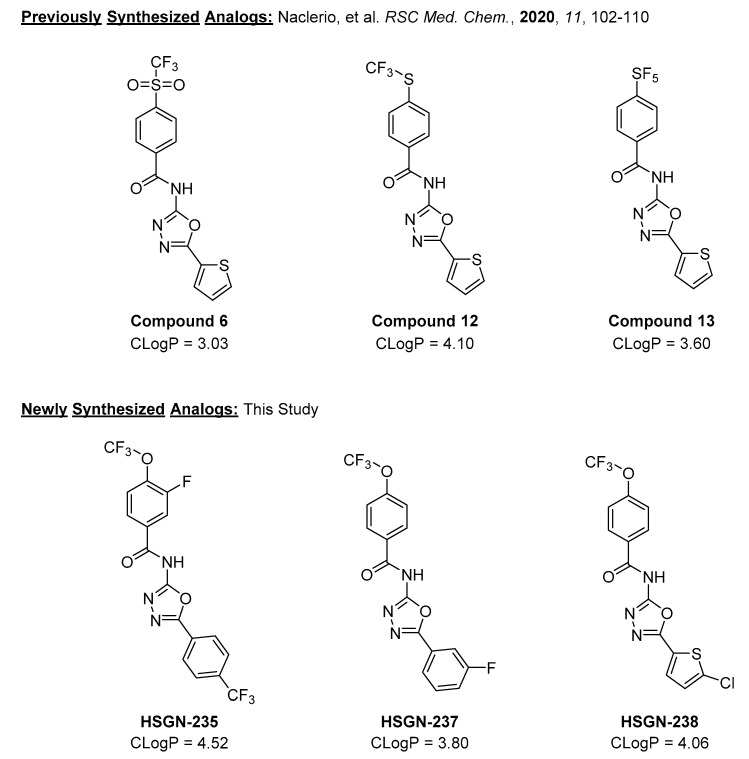
Previously reported analogs as well as newly synthesized N-(1,3,4-oxiadizol-2-yl)benzamides for this study. **Note:** CLogP was calculated using SwissADME.

We previously reported that trifluoromethylsulfonyl (SO_2_CF_3_), trifluoromethylthio (SCF_3_), and pentafluorosulfanyl (SF_5_) containing N-(1,3,4-oxadiazol-2-yl)benzamides (**compounds 6**, **12,** and **13,** respectively) were potent against a panel of drug-resistant Gram-positive bacteria [17]. We wondered if these compounds would be active against N. gonorrhoeae and discovered that **compounds 6**, **12**, and **13** have quite potent activity against N. gonorrhoeae strain 181, with minimum inhibitory concentrations (MICs) of 0.5, 0.06, and 0.06 µg/mL, respectively (see Table 1). While all three compounds have favorable CLogP values, they also contain an unsubstituted thiophene moiety (Figure 1) which can cause toxicity. For example, the cytochrome P450-mediated oxidation of thiophene moeities can lead to reactive metabolites such as thiophene epoxides [18], thiophene-S oxides [18,19], and sulphenic acids [20], which can react with nucleophiles such as glutathione and/or water [21]. However, since **Compounds 6**, **12**, and **13** have shown excellent activities against N. gonorrhoeae as well as adequate CLogP values, we desired to further optimize these compounds via the synthesis of new analogs. We proceeded to use computational methods to guide our synthetic strategy. We began to substitute the benzamide ring with the trifluoromethoxy (OCF_3_) group due to its importance in medicinal chemisty [22,23]. For instance, it was reported that the electronegativity of the OCF_3_ group allows for enhanced in vivo uptake and transport in biological systems [22]. Thus, utilizing this strategy has led to the synthesis of **HSGN-235**, which contained a fluoro atom ortho to the OCF_3_ group as well as a trifluoromethyl phenyl. Yet, **HSGN-235** was found to contain a much larger CLogP value compared to previously synthesized analogs (Figure 1). Since LogP shows a positive correlation between low aqueous solubility and compromising bioavailability (an extremely important attribute when creating antibacterial agents against N. gonorrhoeae) [24], we replaced the thiophene moiety with a substituted thiophene or phenyl group as unsubstituted thiophene could be a toxicophore, as mentioned above. Considering that the addition of halogens to compounds has been shown to improve drug properties and metabolic stability [25,26,27,28,29], our new analogs were made up of compounds with halogen substitutions to a phenyl ring (Figure 1).

**Scheme 1:**
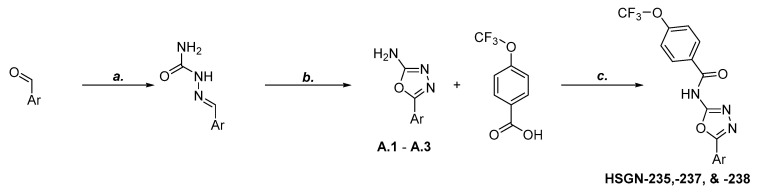
Synthesis of N-(1,3,4-oxadiazol-2-yl)benzamides. Reagents and Conditions: (**a**) Semicarbazide hydrochloride, NaOAc, MeOH:H_2_O (1:1), rt, 30 min. (**b**) Bromine, NaOAc, AcOH, 60°C, 1 h. (**c**) BOP Reagent, DIPEA, DMF, rt, 12 h.

The synthesis of these compounds started with a substituted aryl aldehyde, followed by the addition of semicarbazide and sodium acetate to give the corresponding semicarbazone. Then, using bromine and sodium acetate, the semicarbazone was converted into the subsequent aryl 1,3,4-oxadizol-2-amine. Amide coupling between the aryl 1,3,4-oxadiazol-2-amine and 4-trifluoromethoxy benzoic acid using benzotriazol-1-yloxytris(dimethylamino)phosphonium hexafluorophosphate (BOP) reagent gave the desired N-(1,3,4-oxadiazol-2-yl)benzamides (Scheme 1). 

Trifluoromethoxy containing (1,3,4-oxadiazol-2-yl)benzamides with the substitution of the thiophene moiety with a fluorophenyl (**HSGN-237**) or chlorothiophenyl (**HSGN-238**) groups had potent activity against *N. gonorrhoeae* strain 181 with MICs of 0.125 µg/mL (Table 1). Interestingly, the substitution of the 4-trifluoromethoxy phenyl group with a fluorine, as well as the substitution of the thiophene moiety with trifluoromethylphenyl (**HSGN-235**) only had moderate activity when tested against *N. gonorrhoeae* strain 181 (Table 1). Since both **HSGN-237** and **HSGN-238** contained aromatic rings bearing a halogen atom, we speculate that the loss of activity for **HSGN-235** is due to the addition of the fluorine atom ortho to the trifluoromethoxy group (see Figure 1 and Table 1 for comparisons).

After the initial screening against *N. gonorrhoeae* 181, the anti-gonococcal activity of **HSGN-235**, **-237,** and **-238** was explored against a panel drug-resistant pathogenic N. gonorrhoeae strains, including one WHO reference strain (*N. gonorrhoeae* WHO L) which has a well-characterized antibiogram and phenotypic and genetic markers [30]. As depicted in Table 2, **HSGN-237** and **-238** exhibited potent activity against the tested strains, with inhibitions of their growth at concentrations ranging from 0.03 to 0.125 µg/mL. Both were superior to azithromycin and tetracycline against the tested isolates. On the other hand, **HSGN 235** inhibited the growth of the tested strains at concentrations ranging from 1 to 2 µg/mL. Interestingly, the minimum bactericidal concentration (MBC) values of **HSGN235**, **-237,** and **-238** were the same as or one-fold higher than their corresponding MIC values, indicating that the compounds exhibit bactericidal activity against the tested *N. gonorrhoeae* strains.

### 2.2. Antibacterial Activity of N-(1,3,4-oxadiazol-2-yl)benzamides against Other Bacterial Species:

While the focus of these new *N*-(1,3,4-oxadiazol-2-yl)benzamides is towards *N. gonorrhoeae*, we proceeded to test their activity against other Gram-positive and Gram-negative pathogens. Intriguingly, **HSGN-235**, **HSGN-237**, and **HSGN-238** had potent activity against the tested Gram-positive bacterial pathogens. For instance, all three compounds had potent activity against the staphylococcal strains, with MICs ranging from 0.25 to 1 µg/mL (Table 3). Furthermore, **HSGN-235**, **HSGN-237**, and **HSGN-238** maintained potent activity against clinically relevant Gram-positive bacterial pathogens such as vancomycin-resistant enterococci (VRE) and *Listeria monocytogenes* (Table 3). Additionally, we moved to test if **HSGN-235**, **HSGN-237**, and **HSGN-238** were active against other Gram-negative bacterial pathogens. These compounds were found to be inactive against *E. coli* BW25113. This lack of activity against Gram-negative bacteria appears to be due to **HSGN-235, HSGN-237,** and **HSGN-238** being a substrate for efflux. This can be seen by the shift in the MICs observed for **HSGN-235, HSGN-237,** and **HSGN-238** against wild-type *E. coli* BW25113 (MIC >8 µg/mL for all compounds; Table 3) in comparison to a mutant strain (*E. coli* JW55031), where the AcrAB-TolC multidrug-resistant efflux pump is knocked out (MIC for **HSGN-235, HSGN-237,** and **HSGN-238** improves to 4, 0.25, and 0.06 µg/mL, respectively; Table 3). A similar result was observed with linezolid, an antibiotic known to be a substrate for the AcrAB-TolC efflux pump in Gram-negative bacteria, as reported in previous reports [31,32]. Interestingly, **HSGN-235**, **HSGN-237**, and **HSGN-238** appeared to be bacteriostatic agents, as their MBCs were more than three-fold higher than their corresponding MICs against the tested bacterial strains (Table 3). 

### 2.3. Antibacterial Activity of N-(1,3,4-oxadiazol-2-yl)benzamides against N. gonorrhoeae in Presence of Serum:

An increase in MIC due to antibiotics being highly protein-bound has been documented in several classes of antibiotics [33,34,35]. Therefore, we evaluated our compounds’ activity against *N. gonorrhoeae* in the presence of different concentrations of fetal bovine serum (FBS). As presented in Table S2, the activity of **HSGN-235**, **-237**, and **-238** was reduced in the presence of FBS. The addition of 1%, 5%, and 10% FBS to the media increased the MIC of **HSGN-237** to 0.125, 1, and 4 µg/mL, respectively (see Table S2). Similarly, the MIC of **HSGN-238** also changed to 0.25 and 1 µg/mL (still considered good potency) in the presence of 1% and 5% FBS, respectively, but stayed at 1 µg/mL with the addition of 10% FBS (Table S2). The MIC of **HSGN-235** did not change in the presence of 1% FBS, but increased to 4 and 8 µg/mL in the presence of 5% and 10% FBS, respectively (Table S2). Hydrophobic antibiotics such as antimicrobial peptides and lipopeptides have been found to show an increase in MIC upon the addition of serum to media [36,37]. We predict that the hydrophobicity of these *N*-(1,3,4-oxadiazol-2-yl)benzamides contributes to the rise in MIC in the presence of FBS. Future studies will attempt to develop analogs thereof with fewer protein-binding properties.

### 2.4. N-(1,3,4-Oxadiazol-2-yl)benzamides Are Highly Tolerable to Human Cell Lines:

**Figure 2:**
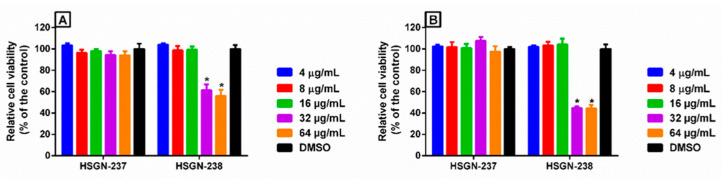
In vitro cytotoxicity assessment of **HSGN-237** and **-238** (tested in triplicate) against human colorectal cells (Caco-2) after (**A**) 24 h, and **(B**) 48 h, using the 3-(4,5-dimethylthiazol-2-yl)-5-(3-carboxymethoxyphenyl)-2-(4-sulfophenyl)-2H-tetrazolium (MTS) assay. Results are presented as percent viable cells relative to DMSO (negative control). Error bars represent standard deviation values. A two-way ANOVA, with post hoc Dunnet’s multiple comparisons test, determined the statistical difference between the values obtained for the compound and DMSO. Asterisks denote statistically significant differences between treatments of cells with either **HSGN-237** or **-238** as compared to DMSO-treated cells. The experiment was repeated 3 independent times.

Prokaryotic cell selectivity is highly important for an antibiotic candidate. Therefore, since **HSGN-237** and **-238** were found to be the most potent analogs against *N. gonorrhoeae*, they were assessed for toxicity to mammalian cells over 24- and 48-h periods (Figure 2A,B). Both compounds showed excellent safety profiles against human colorectal cells (Caco-2). For instance, **HSGN-237** was non-toxic at concentrations higher than 64 µg/mL, which is 512-times higher than the compound‘s corresponding MIC values against *N. gonorrhoeae* (Figure 2A,B). Additionally, **HSGN-238** was non-toxic at concentrations of up to 16 µg/mL which is 128 times higher than the compound‘s corresponding MIC values against *N. gonorrhoeae* (Figure 2A,B).

### 2.5. HSGN-238 Demonstrates High Intestinal Permeability:

Oral bioavailability is a highly important consideration when developing bioactive molecules as therapeutic agents [38]. A critical factor of oral bioavailability is human intestinal absorption. The Caco-2 bidirectional permeability assay is the most widely used in vitro model for predicting if a bioactive molecule can have adequate systemic absorption [39,40]. Thus, we selected **HSGN-238** to act as a model to analyze the drug-like properties of newly synthesized *N*-(1,3,4-oxadiazol-2-yl)benzamides. The assay demonstrated that **HSGN-238** showed an outstanding ability to permeate across Caco-2 bilayers (apparent permeability, P_app_ = 82.3 × 10^−6^ cm s^−1^ from the apical to basolateral and P_app_ = 32.9 × 10^−6^ cm s^−1^ from the basolateral to apical; see Table 4). This permeability is comparable to propranolol (P_app_ = 37.2 × 10^−6^ cm s^−1^ from the apical to basolateral and P_app_ = 22.7 × 10^−6^ cm s^−1^ from the basolateral to apical (Table 4)), a drug that is known to have a high permeability across Caco-2 bilayers. Ranitidine was used as a low-permeability control, as its P_app_ = 0.5 × 10^−6^ cm s^−1^ from the apical to basolateral and P_app_ = 1.3 × 10^−6^ cm s^−1^ from the basolateral to apical. Therefore, the Caco-2 permeability results indicate that **HSGN-238** has a high potential to be strongly absorbed after being administered orally.

## 3. Materials and Methods

### 3.1. Chemistry

General considerations: All reagents and solvents were purchased from commercial sources. The ^1^H, ^13^C, and ^19^F NMR spectra were acquired in DMSO-*d*_6_ as solvent using a 500 MHz spectrometer with Me_4_Si as an internal standard. Chemical shifts are reported in parts per million (δ) and were calibrated using residual undeuterated solvent as an internal reference. Data for ^1^H NMR spectra are reported as follows: chemical shift (δ ppm) (multiplicity, coupling constant (Hz), integration). Multiplicities are reported as follows: s = singlet, d = doublet, t = triplet, q = quartet, m = multiplet, or combinations thereof. High resolution mass spectra (HRMS) were obtained using electron spray ionization (ESI) technique and as TOF mass analyzer. New compounds were characterized by ^1^H NMR, ^13^C NMR, ^19^F NMR, and HRMS data.

### 3.2. Synthesis of 1,3,4-Oxadiazol-2-Amines [A.1-A.3]

The synthesis of A.1-A.3 was performed following a literature-reported procedure [41]. ^1^H, ^13^C, and ^19^F NMR spectra were in agreement with the literature-reported data.

### 3.3. Amide Coupling Procedure for Synthesis of Compounds

A 20 mL screw-capped vial, charged with the corresponding acid (1 eq.), amine (1 eq.), BOP reagent (2.7 eq.), and diisopropylethylamine (23 eq.) in DMF solvent (3 mL), was stirred at room temperature for 16 h. After completion, the reaction mixture was concentrated under reduced pressure, followed by flash column chromatography (hexanes:ethyl acetate 80:20 to 60:40) to give the desired product.

### 3.4. 3-Fluoro-4-(trifluoromethoxy)-N-(5-(4-(trifluoromethyl)phenyl)-1,3,4-oxadiazol-2-yl)benzamide (**HSGN-235**)

Off-white solid (34 mg, 18%). ^1^H NMR (500 MHz, DMSO-*d*_6_) δ 8.1 (m, 2H), 8.0 (m, 2H), 7.8 (m, 2H), 7.5 (m, 1H). ^13^C NMR (126 MHz, DMSO-*d*_6_) δ 161.9, 160.1, 158.4, 153.0 (d, *J* = 258.3 Hz), 136.3 (d, *J* = 12.6 Hz), 132.0 (q, *J* = 32.8 Hz), 129.7, 127.5, 127.4, 126.9 (d, *J* = 3.78 Hz), 126.8, 126.0 (d, *J* = 5.04 Hz), 125.3, 123.1 (q, *J* = 288.5 Hz), 121.5 (q, *J* = 259.6 Hz). ^19^F NMR (471 MHz, DMSO-*d*_6_) δ −59.0 (s, 3F), −62.9 (s, 3F), −132.1 (s, 1F). HRMS (ESI) *m/z* calcd for C_17_H_9_F_7_N_3_O_3_ [M + H]^+^ 436.0532, found 436.0531.

### 3.5. N-(5-(3-Fluorophenyl)-1,3,4-oxadiazol-2-yl)-4-(trifluoromethoxy)benzamide (**HSGN-237**)

Off-white solid (42 mg, 24%). ^1^H NMR (500 MHz, DMSO-*d*_6_) δ 8.2 (dd, *J* = 8.5, 3.6 Hz, 2H), 7.8 (dd, *J* = 7.9, 3.6 Hz, 1H), 7.7 – 7.6 (m, 2H), 7.5 (ddd, *J* = 34.6, 10.1, 6.0 Hz, 3H). ^13^C NMR (126 MHz, DMSO-*d*_6_) δ 165.0, 163.7 (d, *J* = 245.7 Hz), 160.2, 158.8, 151.9, 132.4 (d, *J* = 8.82 Hz), 132.2, 131.3, 125.9 (d, *J* = 8.82 Hz), 122.8, 121.5 (q, *J* = 259.6 Hz), 121.1, 119.3 (d, *J* = 21.4 Hz), 113.3 (d, *J* = 23.9 Hz). ^19^F NMR (471 MHz, DMSO-*d*_6_) δ −57.8 (s, 3F), −112.5 (q, *J* = 8.1 Hz, 1F). HRMS (ESI) *m/z* calcd for C_16_H_10_F_4_N_3_O_3_ [M + H]^+^ 368.0658, found 368.0659.

### 3.6. N-(5-(5-Chlorothiophen-2-yl)-1,3,4-oxadiazol-2-yl)-4-(trifluoromethoxy)benzamide (**HSGN-238**)

Off-white solid (45 mg, 24%). ^1^H NMR (500 MHz, DMSO-*d*_6_) δ 8.1 (d, *J* = 8.5 Hz, 2H), 7.6 (d, *J* = 4.1 Hz, 1H), 7.5 (m, 2H), 7.3 (m, 1H). ^13^C NMR (126 MHz, DMSO-*d*_6_) δ 164.8, 158.0, 156.5, 151.9, 133.5, 131.9, 131.3, 129.9, 129.1, 123.7, 121.4 (q, *J* = 258.3 Hz), 121.1. ^19^F NMR (471 MHz, DMSO-*d*_6_) δ -57.8 (s, 3F). HRMS (ESI) *m/z* calcd for C_14_H_8_ClF_3_N_3_O_3_S [M + H]^+^ 389.9927, found 389.9925.

### 3.7. Bacterial Strains, Media, Reagents and Cell Lines

*Neisseria gonorrhoeae* clinical isolates (Table S1) used in this study were obtained from the CDC. *S. aureus*, MRSA, *E. faecalis*, *E. faecium*, and *L. monocytogenes* strains were obtained from the American Type Culture Collection (ATCC). *E. coli* BW25113 and JW25113 were obtained from the Coli Genetic Stock Center (CGSC), Yale University, USA. Media and reagents were purchased from commercial vendors: Brucella broth, chocolate II agar, cation-adjusted Mueller Hinton broth, tryptic soy broth (TSB), and tryptic soy agar (TSA) (Becton, Dickinson and Company, Cockeysville, MD, USA); yeast extract and dextrose (Fisher Bioreagents, Fairlawn, NJ, USA), proteose-peptone, nicotinamide adenine dinucleotide (NAD), agarose and tetracycline (Sigma-Aldrich, St. Louis, MO, USA); hematin, Tween 80, pyridoxal, linezolid and gentamicin sulfate (Chem-Impex International, Wood Dale, IL, USA); Dulbecco’s Modified Eagle Medium (DMEM), fetal bovine serum (FBS) and phosphate-buffered saline (PBS) (Corning, Manassas, VA, USA); and azithromycin (TCI America, Portland, OR, USA). Human colorectal adenocarcinoma epithelial cells (Caco-2) (ATCC HTB-37) was obtained from the American Type Culture Collection (ATCC) (Manassas, VA, USA). Compounds were synthesized from commercial sources in our laboratory.

### 3.8. Determination of the MICs of Compounds and Control Drugs against N. gonorrhoeae Strains

The MICs of the tested compounds and control drugs; azithromycin, and tetracycline were determined using the broth microdilution as described previously [42,43,44]. Briefly, bacteria were grown overnight on chocolate agar II at 37° C in the presence of 5% CO_2_. Afterwards, a bacterial suspension equivalent to 1.0 McFarland standard was prepared and diluted in brucella broth supplemented with yeast extract, neopeptone, hematin, pyridoxal, and NAD. Test agents were added in the first row of the 96-well plates and serially diluted along the plates. Plates were then incubated at 37° C in the presence of 5% CO_2_ for 24 h. The MICs reported in Table 1 are the minimum concentrations of the compounds and control drugs that could completely inhibit the visual growth of bacteria. The minimum bactericidal concentration (MBC) of these drugs was tested by plating 4 µL from wells with no growth onto chocolate agar II plates. Plates were then incubated at 37 ° C in the presence of 5% CO_2_ for 24 h. The MBC was categorized as the lowest concentration that reduced bacterial growth by 99.9% [45,46,47]. 

### 3.9. Determination of the MICs and MBCs of Compounds and Control Drugs against Clinically Important Gram-Positive and Gram-Negative Bacteria 

The minimum inhibitory concentrations (MICs) of the tested compounds and control drugs, linezolid, vancomycin, and gentamicin, were determined using the broth microdilution method according to the guidelines outlined by the Clinical and Laboratory Standards Institute (CLSI) [48] against clinically relevant bacterial (*Staphylococcus aureus*, MRSA, *Escherichia coli*, *Enterococcus faecalis* and *Enterococcus faecium* strains. *S. aureus*, MRSA, *E. coli, Enterococcus faecalis* and *Enterococcus faecium* were grown aerobically overnight on tryptone soy agar (TSA) plates at 37° C. Afterwards, a bacterial solution equivalent to 0.5 McFarland standard was prepared and diluted in cation-adjusted Mueller–Hinton broth (CAMHB) (for *S. aureus*, MRSA, and *E. coli*) to achieve a bacterial concentration of about 5 × 10^5^ CFU/mL. *Enterococcus faecalis* and *Enterococcus faecium* 0.5 McFarland standard solution was diluted in tryptone soy broth (TSB) to achieve a bacterial concentration of about 5 × 10^5^ CFU/mL. Compounds and control drugs were added in the first row of 96-well plates and serially diluted with the corresponding media containing bacteria. Plates were then incubated as previously described. MICs reported in Table 2 are the minimum concentration of the compounds and control drugs that could completely inhibit the visual growth of bacteria. The minimum bactericidal concentration (MBC) was tested by spotting 4 µL from wells with no growth onto TSA plates. Plates were incubated at 37 ° C for at least 18 h before recording the MBC. The MBC was categorized as the lowest concentration that reduced bacterial growth by 99.9% [45,46,47]. 

### 3.10. In Vitro Cytotoxicity Analysis of **HSGN-237** and **-238** against Human Colorectal Cells

**HSGN-237** and **-238** were assayed for potential cytotoxicity against a human colorectal adenocarcinoma (Caco-2) cell line, as described previously [16,49,50]. Briefly, tested compounds were incubated with caco-2 cells for 24 and 48 h. Then, the cells were incubated with MTS (3-(4,5-dimethylthiazol-2-yl)-5-(3-carboxymethoxyphenyl)-2-(4-sulfophenyl)-2H-tetrazolium) reagent for 4 h before measuring the absorbance values (OD_490_).

### 3.11. Caco-2 Permeability Assay

Assay and data analysis were performed by Eurofins Panlabs (MO, USA) according to a previously reported protocol [51,52]. The apparent permeability coefficient (P_app_) of the tested agents was calculated using the equation below:

$$P_{app}(\mathrm{cm}/s)=\frac{V_{R}*C_{R,end}}{\Delta t}*\frac{1}{A*\left(C_{D,mid}-C_{R,mid}\right)},$$

where V_R_ is the volume of the receiver chamber. C_R,end_ is the concentration of the test compound in the receiver chamber at the end time point, Δt is the incubation time, and A is the surface area of the cell monolayer. C_D,mid_ is the calculated mid-point concentration of the test compound in the donor side, which is the mean value of the donor concentration at 0 minutes and the donor concentration at the end time point. C_R,mid_ is the mid-point concentration of the test compound in the receiver side, which is one half of the receiver concentration at the end time point. Concentrations of the test compound were expressed as peak areas of the test compound.

## 4. Conclusions

We have identified promising *N*-(1,3,4-oxadiazol-2-yl)benzamides with potent antibacterial activity against *N. gonorrhoeae*. Furthermore, **HSGN-237** and **-238** exhibited ha ighly acceptable tolerability to human colon cells. Moreover, when assessed using a Caco-2 bidirectional permeability assay, **HSGN-238** showed a remarkable ability to cross Caco-2 bilayers, indicating that it would have favorable systemic absorption. Thus, the potent antibacterial profiles of these *N*-(1,3,4-oxadiazol-2-yl)benzamides warrants further investigation and exploration as potential therapeutics to treat drug-resistant *N. gonorrhoeae* infections. OCF_3_-modified *N*-(1,3,4-oxadiazol-2-yl)benzamides can be added to the list of novel antibacterial agents with novel scaffolds that we have reported [53,54,55,56].

## Figures and Tables

**Table 1:** MICs (µg/mL) of the previously reported analogs (**compounds 6**, **12,** and **13**) and the new compounds (**HSGN-235**, **-237,** and **-238**) against *N. gonorrhoeae* strain 181. The experiment was repeated 3 independent times and the same MIC values were obtained. Compounds tested are in bold while control drugs are in regular script.

**Compound/**Control Drug	***N. gonorrhoeae* Strain 181**
**Compound 6**	0.5
**Compound 12**	0.06
**Compound 13**	0.06
**HSGN-235**	16
**HSGN-237**	0.125
**HSGN-238**	0.125
Azithromycin	256
Tetracycline	2

**Table 2:** MICs and MBCs (µg/mL) of **HSGN-235**, **-237,** and **-238** against *N. gonorrhoeae* clinical isolates. The experiment was repeated three independent times and the same MIC values were obtained.

Bacterial Strains	HSGN-235		HSGN-237		HSGN-238		Azithromycin		Tetracycline	
	MIC	MBC	MIC	MBC	MIC	MBC	MIC	MBC	MIC	MBC
*N. gonorrhoeae* 165	2	2	0.06	0.125	0.125	0.25	1	4	4	8
*N. gonorrhoeae* 166	2	2	0.06	0.06	0.125	0.25	0.5	1	2	8
*N. gonorrhoeae* 194	1	1	0.03	0.06	0.125	0.125	0.25	0.5	1	4
*N. gonorrhoeae* 197	1	2	0.03	0.06	0.125	0.125	0.5	2	2	4
*N. gonorrhoeae* 200	2	2	0.06	0.06	0.125	0.125	0.5	0.5	2	8
*N. gonorrhoeae* WHO L	1	2	0.06	0.06	0.06	0.125	0.5	1	0.5	2

**Table 3:** MICs (µg/mL) and minimum bactericidal concentrations (MBCs, in µg/mL) of **HSGN-235, HSGN-237,** and **HSGN-238** and control drugs (vancomycin, linezolid, and gentamicin) against a panel of clinically important Gram-positive and Gram-negative bacterial pathogens including: *Staphylococcus aureus*, methicillin-resistant *Staphylococcus aureus* (MRSA)*, Enterococcus faecalis*, *Enterococcus faecium, Listeria monocytogenes*, and *Escherichia coli.* The experiment was repeated three independent times and the same MIC values were obtained.

	HSGN-235		HSGN-237		HSGN-238		Vancomycin		Linezolid		Gentamicin	
Bacterial Strains	MIC	MBC	MIC	MBC	MIC	MBC	MIC	MBC	MIC	MBC	MIC	MBC
*S. aureus* ATCC 25923	1	>64	0.25	>64	0.25	>64	1	1	2	64	NT	NT
MRSA USA300	**0.5**	64	0.25	32	0.25	16	1	2	1	16	NT	NT
*E. faecalis* ATCC 29212	4	32	1	>64	1	32	1	1	2	64	NT	NT
*VRE. faecalis*ATCC 51575	2	>64	1	>64	1	32	>64	>64	2	64	NT	NT
*VRE. faecalis* ATCC 51299	1	64	0.5	16	0.25	8	>64	>64	1	32	NT	NT
*VRE. faecium* ATCC 700221	1	32	0.5	8	0.25	8	32	32	2	64	NT	NT
*L. monocytogenes* ATCC 19115	1	64	0.5	64	0.5	32	1	1	2	64	NT	NT
*E. coli* *BW25113* *(wild-type strain)*	>8	>8	>8	>8	>8	>8	>64	>64	>64	>64	0.25	0.25
*E. coli* *JW55031* *(TolC Mutant)*	4	>64	0.25	16	0.06	32	>64	>64	8	>64	0.25	0.25

NT: Not tested.

**Table 4:** Caco-2 permeability analysis for HSGN-238 and control drugs.

**Compound/**Control Drug	**Mean A → B P_app_ (cm s^−1^)**	**Mean B → A P_app_ (cm s^−1^)**	**Notes**
**HSGN-238**	82.3 × 10^−6^	32.9 × 10^−6^	High Permeability
Ranitidine	0.5 × 10^−6^	1.3 × 10^−6^	Low Permeability Control
Propranolol	37.2 × 10^−6^	22.7 × 10^−6^	High Permeability Control

## Supplementary Materials

The following are available online at https://www.mdpi.com/1422-0067/22/5/2427/s1.
