# Supplementary material for: N-(1,3,4-Oxadiazol-2-yl)Benzamides as Antibacterial Agents against Neisseria gonorrhoeae
Source: Int J Mol Sci. 2021 Feb 28;22(5):2427. doi: 10.3390/ijms22052427 (PMC7957578; doi:10.3390/ijms22052427)
Supplement: Supplementary file 1 [file ijms-22-02427-s001.pdf]

Supplementary Information:

N-(1,3,4-oxadiazol-2-yl)benzamides as antibacterial agents against *Neisseria gonorrhoeae*

George A. Naclerio [1], Nader S. Abutaleb [2], Marwa Alhashimi [2], Mohamed N. Seleem [2,3,4], and Herman O. Sintim [1,4,]

[1] Chemistry Department, Institute for Drug Discovery, Purdue University, West Lafayette, IN 47907, United States; gnacleri@purdue.edu (G.A.N.); hsintim@purdue.edu (H.O.S.)
[2] Department of Comparative Pathobiology, Purdue University College of Veterinary Medicine, West Lafayette, Indiana 47907, United States; nabutale@purdue.edu (N.S.A.); alhashim@purdue.edu (M.A); mseleem@purdue.edu (M.N.S.)
[3] Department of Biomedical Sciences and Pathobiology, Virginia-Maryland College of Veterinary Medicine, Virginia Polytechnic Institute and State University, Blacksburg, VA 24060, United States; seleem@vt.edu (M.N.S)
[4] Purdue Institute of Inflammation, Immunology, and Infectious Disease, West Lafayette, Indiana 47907, United States; mseleem@purdue.edu (M.N.S.); hsintim@purdue.edu (H.O.S.)

Table of Contents:

I. Description of Bacterial Isolates Used for Study

II. Antibacterial activity of *N*-(1,3,4-oxadiazol-2-yl)benzamides against *N. gonorrhoeae* in presence of serum

III. ^{1}H, ^{13}C, and ^{19}F Spectra of Analogs

I. <u>Table S1. Bacterial Isolates Used for Study:</u>

**Bacterial strains**	**Source/Description**
Neisseria gonorrhoeae 181	CDC isolate.<br>Resistant to tetracycline and azithromycin.
Neisseria gonorrhoeae 165	CDC isolate.<br>Resistant to tetracycline, penicillin, and ciprofloxacin.
Neisseria gonorrhoeae 166	CDC isolate.<br>Resistant to tetracycline, penicillin, and ciprofloxacin.
Neisseria gonorrhoeae 194	CDC isolate.<br>Resistant to penicillin, not susceptible to ceftriaxone, cefixime and cefpodoxime.
Neisseria gonorrhoeae 197	CDC isolate.<br>Resistant to tetracycline, penicillin, and ciprofloxacin.
Neisseria gonorrhoeae 200	CDC isolate.<br>Resistant to tetracycline, penicillin, and ciprofloxacin.
Neisseria gonorrhoeae WHO L	WHO reference strain.<br>Isolated in Asia, 1996.<br>Resistant to tetracycline, penicillin, and ciprofloxacin.
Staphylococcus aureus ATCC 25923	Clinical isolate.<br>Quality control strain for media testing and susceptibility testing.
MRSA NRS384 (USA300)	Isolated from a wound in Mississippi, USA.<br>Community-acquired MRSA strain.<br>Resistant to erythromycin and tetracycline.
Enterococcus faecalis ATCC 29212	Isolated from urine.<br>Quality control strain for food testing, media testing and susceptibility testing.
Enterococcus faecalis ATCC 51575	Resistant to gentamicin, streptomycin, and vancomycin<br>Sensitive to teichoplanin.<br>Positive for *vanB*
Enterococcus faecalis ATCC 51299	Isolated from peritoneal fluid, Missouri, USA.<br>Resistant to vancomycin.<br>Sensitive to Teicoplanin.<br>Positive for *vanB*
Enterococcus faecium ATCC 700221	Isolated from human faeces, Connecticut, USA.<br>Resistant to Vancomycin and Teicoplanin.<br>Positive for *vanA.*
Escherichia coli BW 25113	Obtained from the Coli Genetic Stock Center (CGSC), Yale University, USA.<br>Wild-type strain.
Escherichia coli JW 55031	Obtained from the Coli Genetic Stock Center (CGSC), Yale University, USA.<br>*tolC*-mutant. Deficient in AcrAB-TolC efflux pump.

II. Table S2. MICs (µg/mL) of **HSGN-235**, **-237**, **-238** and control antibiotics against *N. gonorrhoeae* CDC 165 in presence of FBS. The experiment was repeated for 3 independent times.

**Compound/**Control Antibiotic	**No FBS**	**1% FBS**	**5% FBS**	**10% FBS**
**HSGN-235**	2	2	4	8
**HSGN-237**	0.06	0.125	1	4
**HSGN-238**	0.125	0.25	1	1
Azithromycin	1	1	1	2
Tetracycline	4	2	4	4

III. ^{1}H, ^{13}C, and ^{19}F NMR Spectra of Analogs:

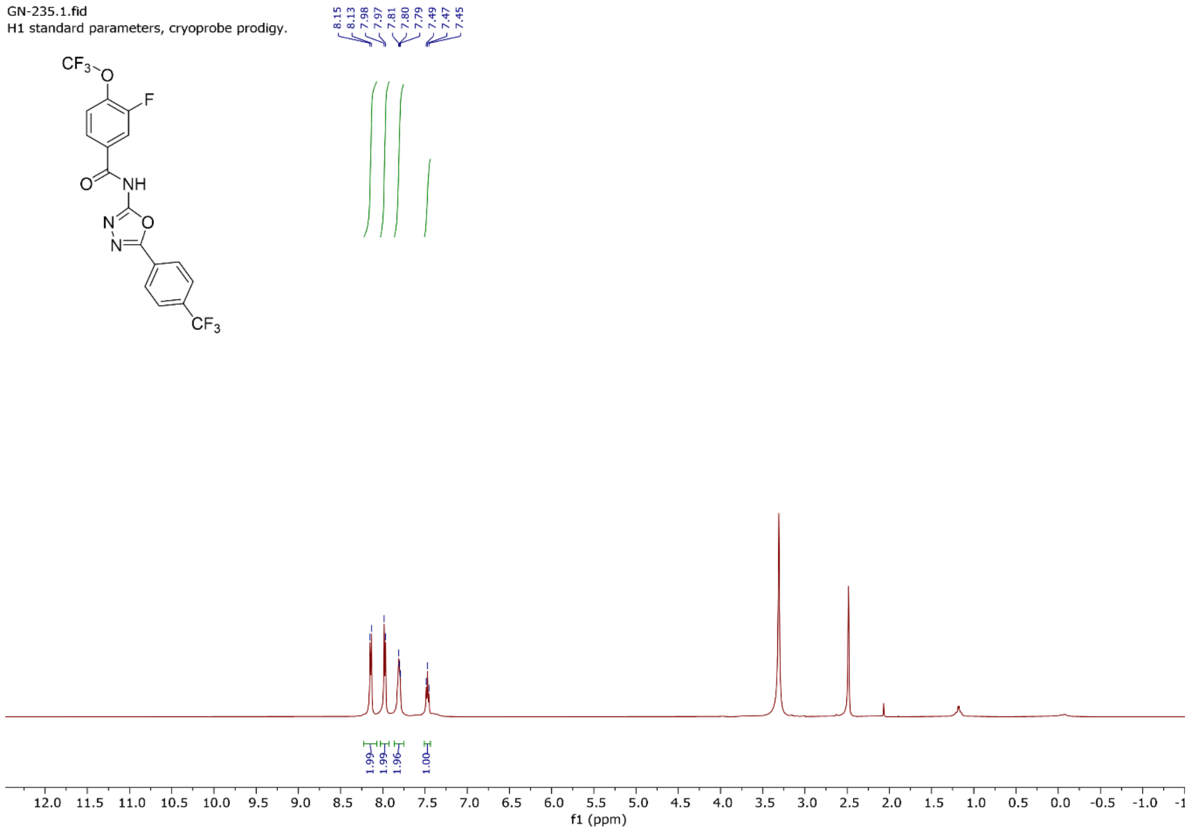

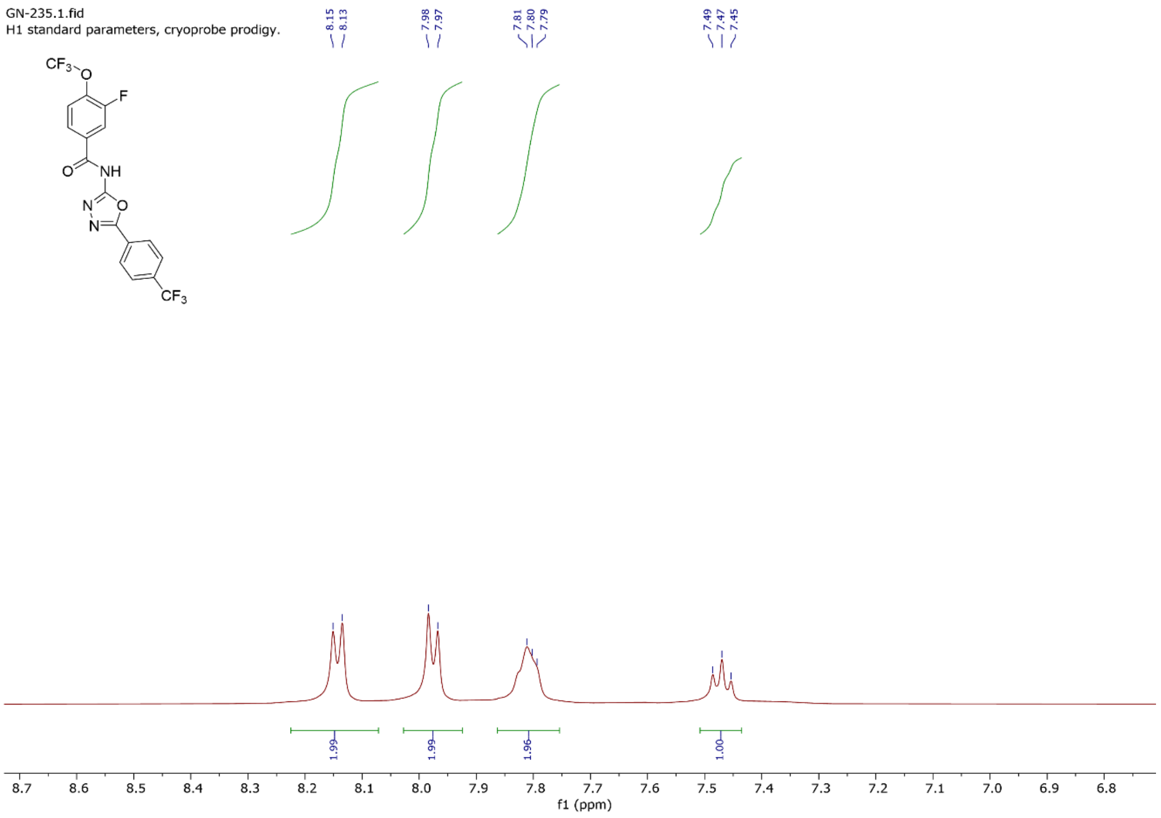

GN-235.2.fid
C13 standard parameters, cryoprobe prodigy. Pulprog = zgig30zr.jsh.

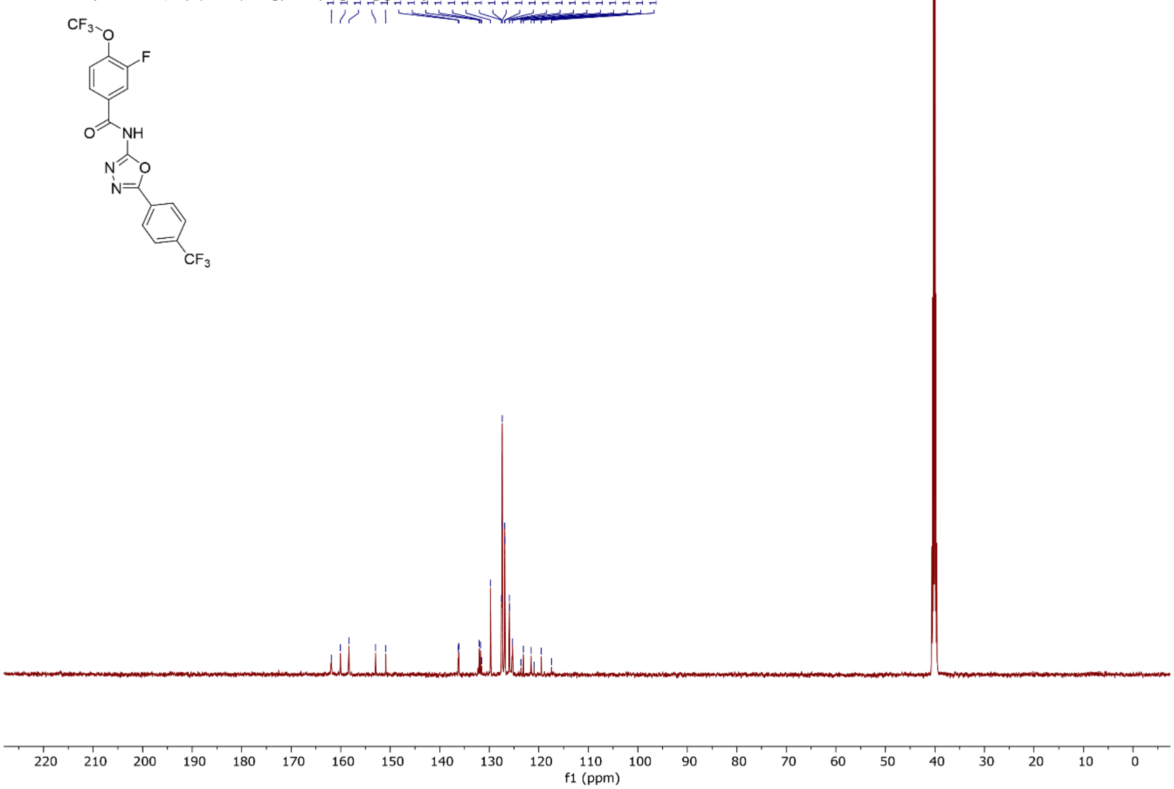

GN-235.3.fid
F19 standard parameters, cryoprobe prodigy.
DE is lengthened compared to default.

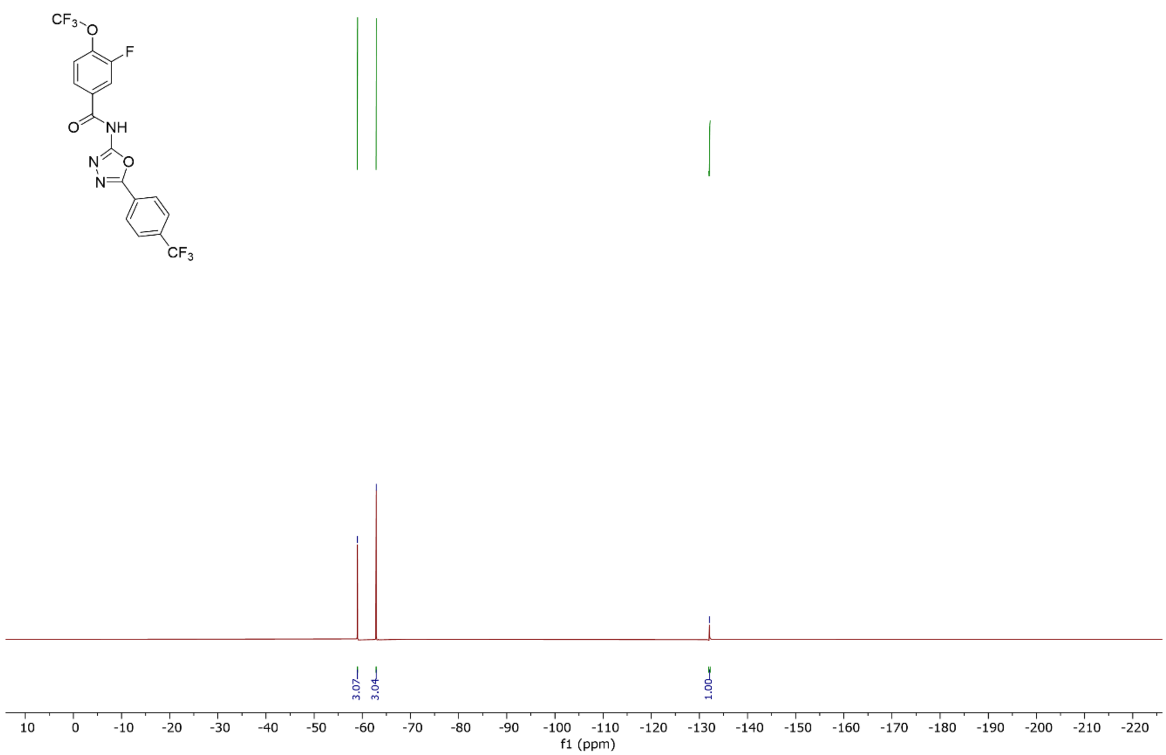

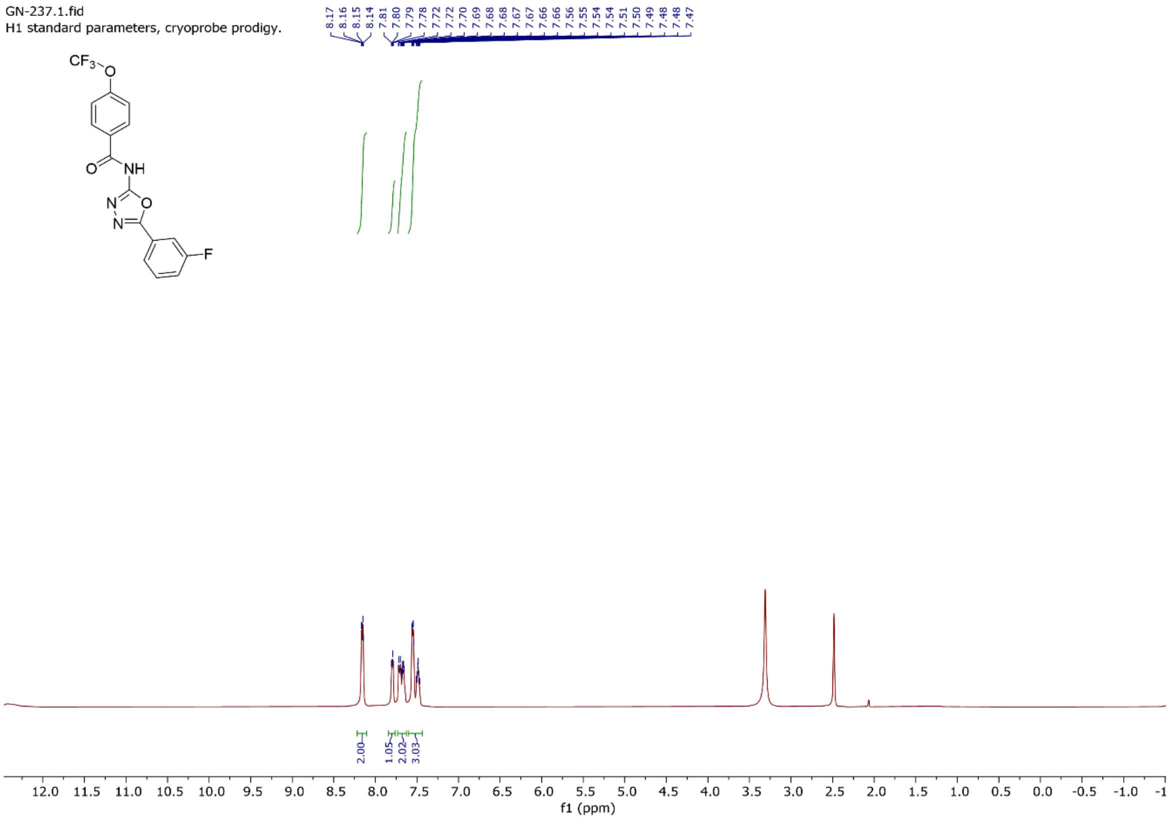
GN-237.1.fid
H1 standard parameters, cryoprobe prodigy.
8.17 8.16 8.15 8.14 7.81 7.80 7.79 7.78 7.72 7.72 7.70 7.69 7.68 7.68 7.67 7.67 7.66 7.66 7.56 7.55 7.54 7.54 7.51 7.50 7.49 7.48 7.48 7.47
2.00 1.05 2.02 3.03
f1 (ppm)

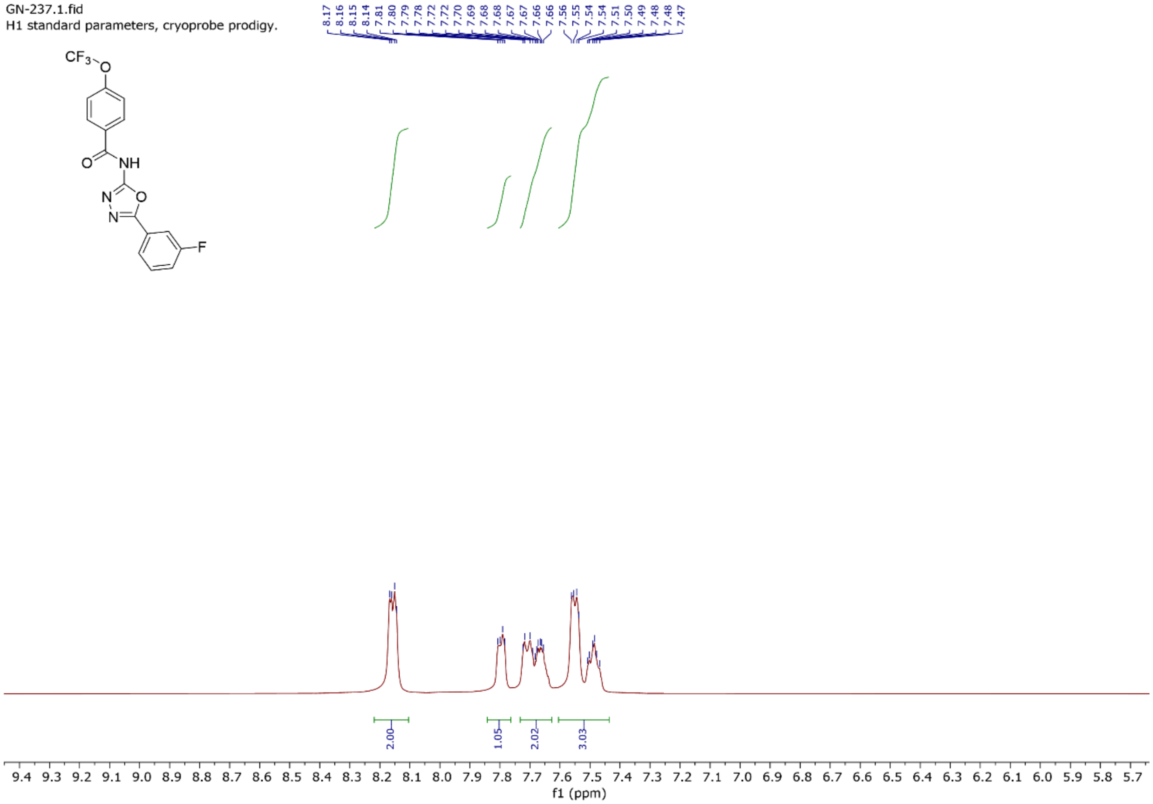
GN-237.1.fid
H1 standard parameters, cryoprobe prodigy.
8.17 8.16 8.15 8.14 7.81 7.80 7.79 7.78 7.72 7.72 7.70 7.69 7.68 7.68 7.67 7.67 7.66 7.66 7.56 7.55 7.54 7.54 7.51 7.50 7.49 7.48 7.48 7.47
2.00 1.05 2.02 3.03
f1 (ppm)

GN-237.2.fid
C13 standard parameters, cryoprobe prodigy. Pulprog = zgig30zr.jsh.

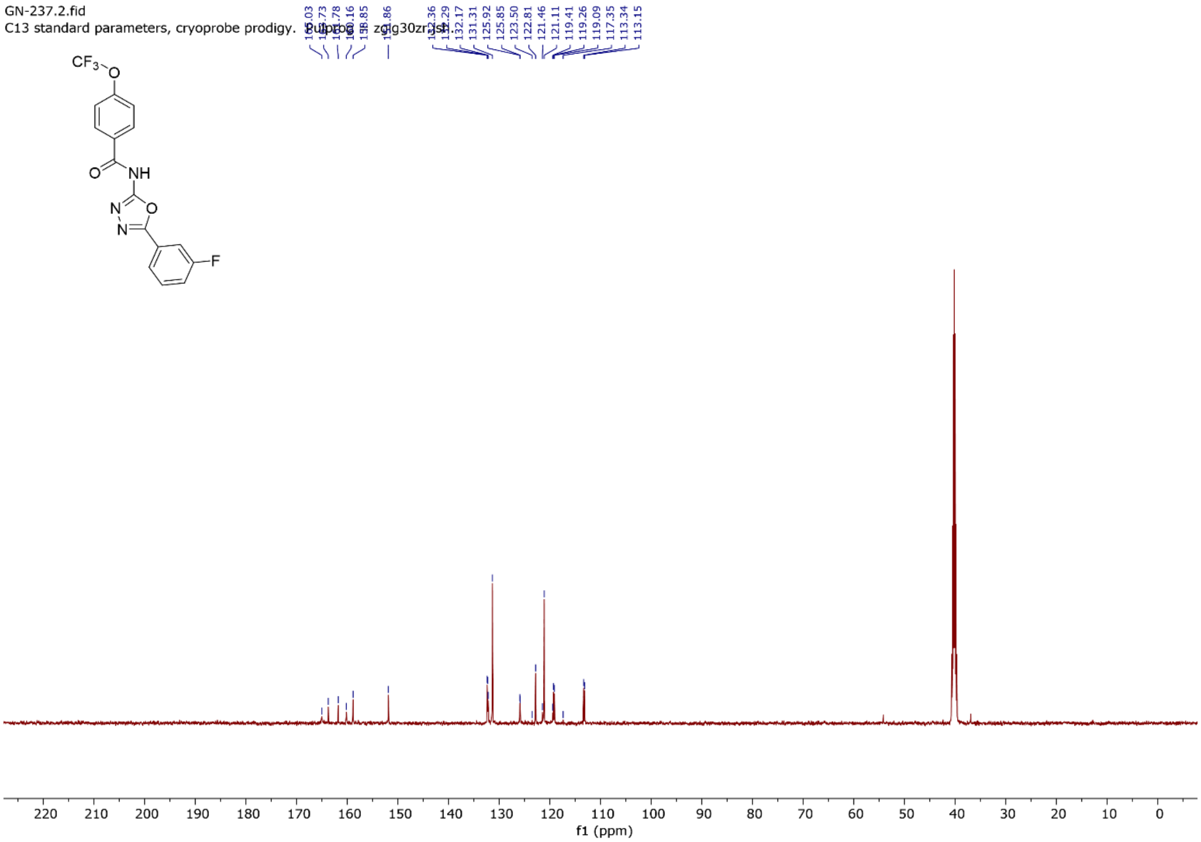

GN-237.3.fid
F19 standard parameters, cryoprobe prodigy.
DE is lengthened compared to default.

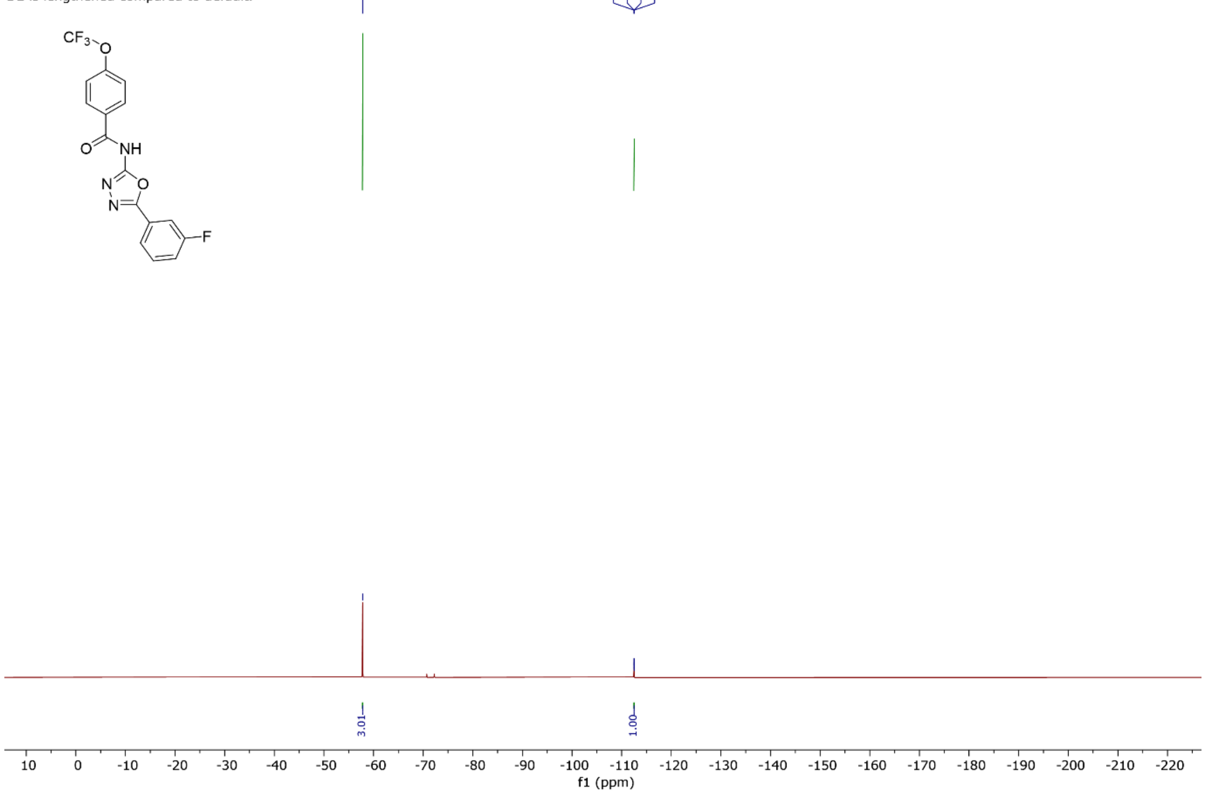

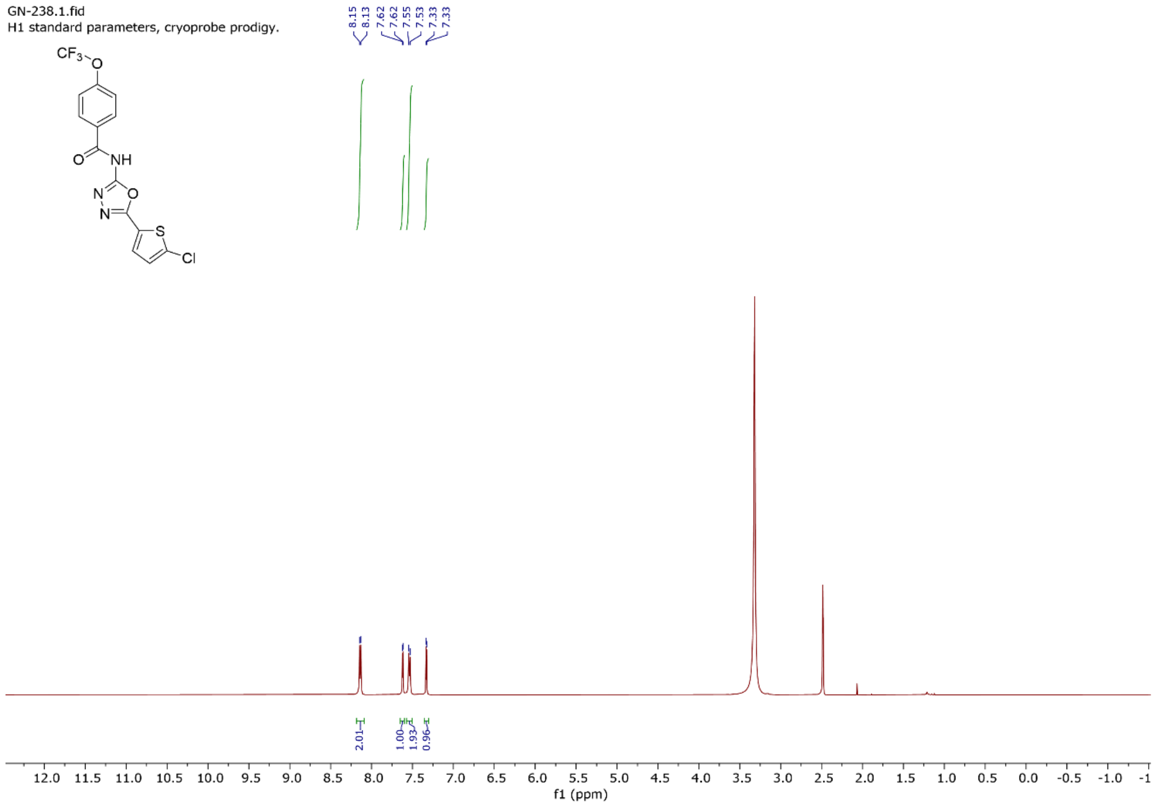
GN-238.1.fid
H1 standard parameters, cryoprobe prodigy.
CF3 O NH N N O S Cl
8.15
8.13
7.62
7.62
7.55
7.53
7.33
7.33
2.01
1.00
1.93
0.96
12.0 11.5 11.0 10.5 10.0 9.5 9.0 8.5 8.0 7.5 7.0 6.5 6.0 5.5 5.0 4.5 4.0 3.5 3.0 2.5 2.0 1.5 1.0 0.5 0.0 -0.5 -1.0
f1 (ppm)

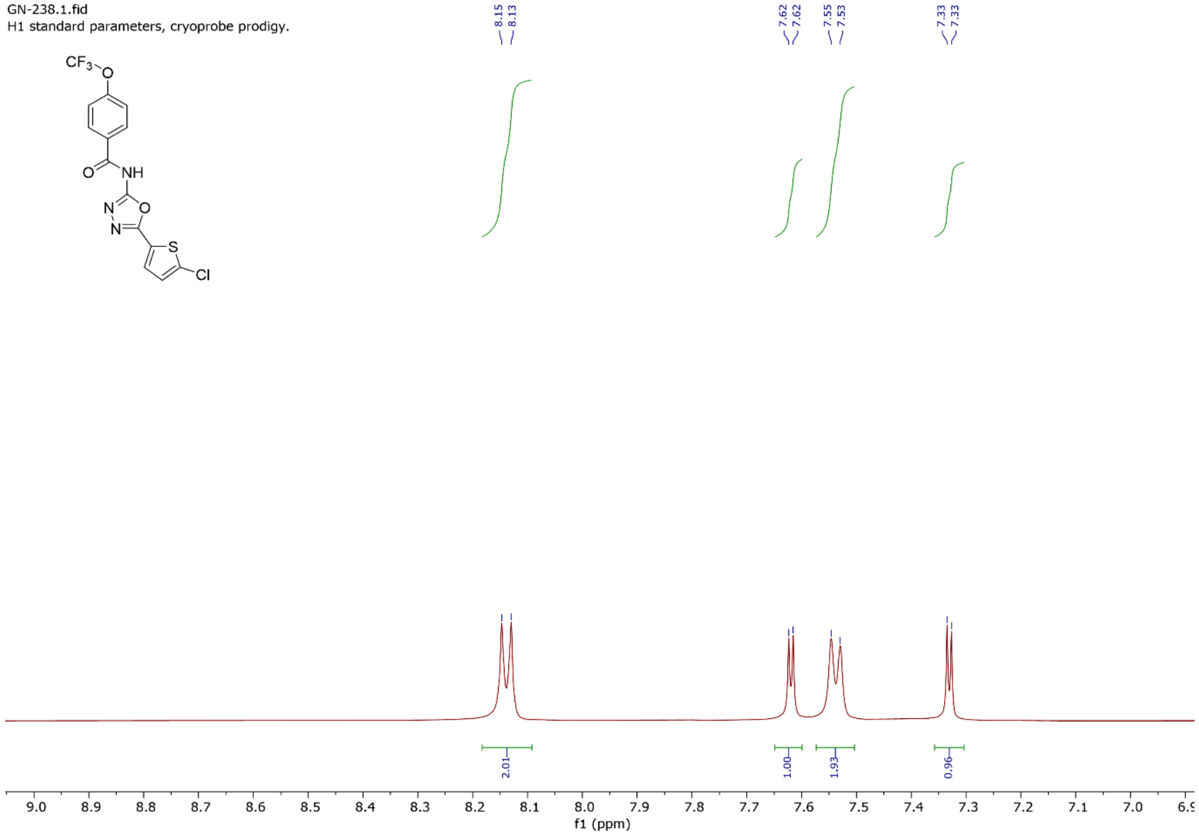
GN-238.1.fid
H1 standard parameters, cryoprobe prodigy.
CF3 O NH N N O S Cl
8.15
8.13
7.62
7.62
7.55
7.53
7.33
7.33
2.01
1.00
1.93
0.96
9.0 8.9 8.8 8.7 8.6 8.5 8.4 8.3 8.2 8.1 8.0 7.9 7.8 7.7 7.6 7.5 7.4 7.3 7.2 7.1 7.0
f1 (ppm)

GN-238.2.fid
C13 standard parameters, cryoprobe prodigy. Pulprog = zgig30zr.jsh

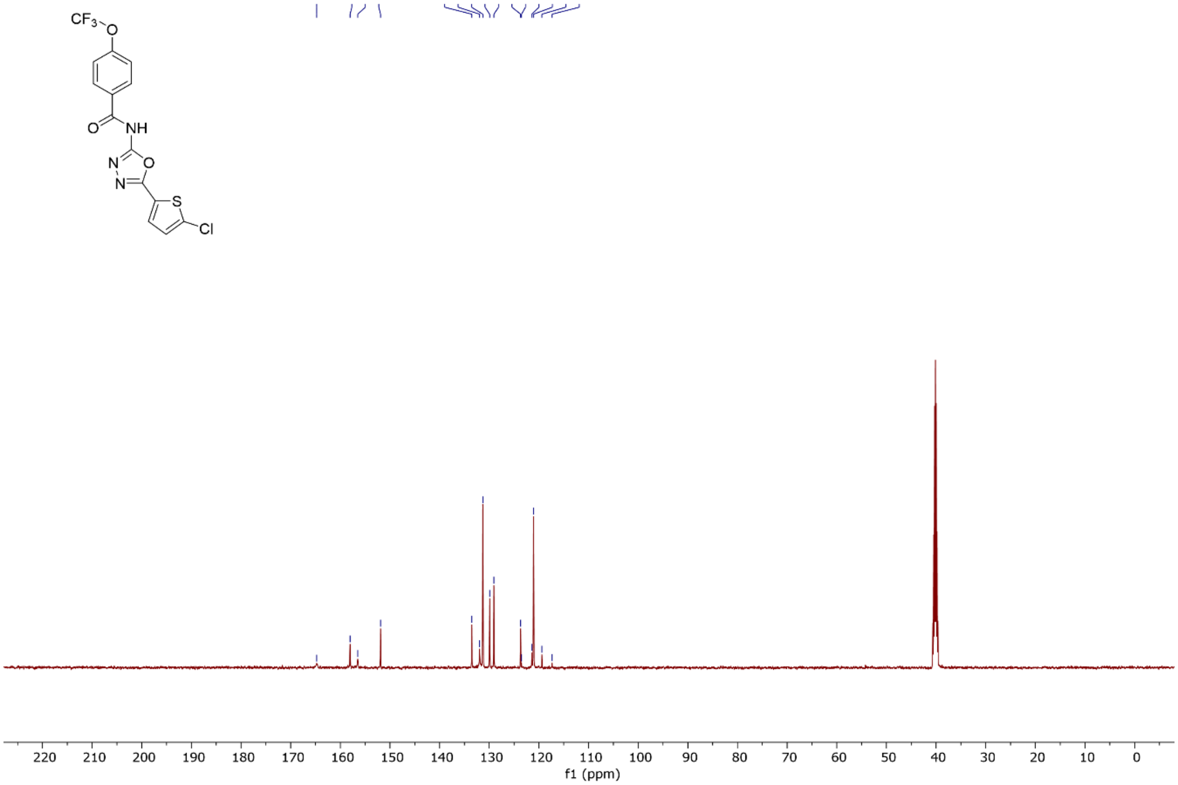

GN-238.3.fid
F19 standard parameters, cryoprobe prodigy.
DE is lengthened compared to default.

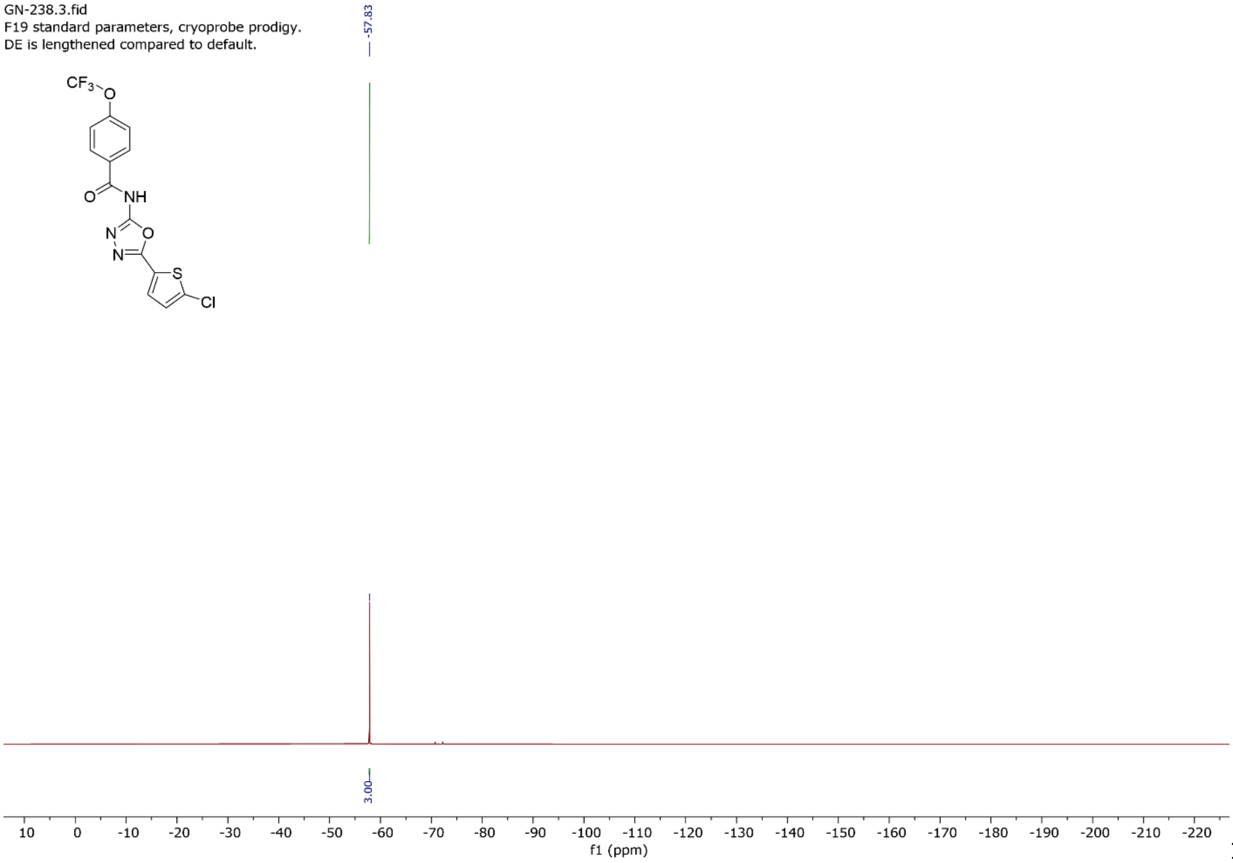

1.
